# Supplementary material for: Deployment of endocytic machinery to periactive zones of nerve terminals is independent of active zone assembly and evoked release
Source: eLife. 2026 Jun 17;14:RP107276. doi: 10.7554/eLife.107276 (PMC13275065; doi:10.7554/eLife.107276)
Supplement: Peer review history

# Editor report: eLife Assessment

**DOI:** 10.7554/eLife.107276.3.sa0

**Authors:** Dion K Dickman

**Affiliations:** University of Southern California United States

**Keywords:** Compelling, Important

This is an **important** and rigorous study that addresses the question of what determines the spatial organization of endocytic zones at synapses. The authors use **compelling** approaches, in both *Drosophila* and rodent model systems, to define the role of activity and active zone structure on the organization of the periactive zone. While the findings are primarily negative, they are carefully executed and contribute to the field by refining existing models of presynaptic organization.

# Referee report: Reviewer #1 (Public review)

**DOI:** 10.7554/eLife.107276.3.sa1

Summary:

In this manuscript, Emperador-Melero et al. seek to determine whether recruitment of endocytic machinery to the periactive zone is activity-dependent or tethered to delivery of active zone machinery. They use genetic knockouts and pharmacological block in two model synapses - cultured mouse hippocampal neurons and *Drosophila* neuromuscular junctions - to determine how well endocytic machinery localizes after chronic inhibition or acute depolarization by super-resolution imaging. They find acute depolarization in both models have minimal to no effect on the localization of endocytic machinery at the periactive zone, suggesting that these proteins are constitutively maintained rather than upregulated in response to evoked activity. Interestingly, chronic inhibition slightly increases endocytic machinery levels, implying a potential homeostatic upregulation in preparation for rebound depolarization. Using genetic knockouts, the authors show that localization of endocytic machinery to periactive zones occurs independently of proper active zone assembly, even in the absence of upstream organizers like Liprin-α.

Overall, they propose that the constitutive deployment of endocytic machinery reflects its critical role in facilitating rapid and reliable membrane internalization during synaptic functions beyond classical endocytosis, such as regulation of the exocytic fusion pore and dense-core vesicle fusion. Although many experiments reveal limited changes in the localization or abundance of endocytic machinery, the findings are thorough, and data substantially supports a model in which endocytic components are organized through a pathway distinct from that of the active zone. This work advances our understanding of synaptic dynamics by supporting a model in which endocytic machinery is constitutively recruited and regulated by distinct upstream organizers compared to active zone proteins. It also highlights the utility of super-resolution imaging across diverse synapse types to uncover functionally conserved elements of synaptic biology.

Strengths:

The study's technical strengths, particularly the use of super-resolution microscopy and rigorous image analyses developed by the group, bolster their findings.

Weaknesses:

One limitation, acknowledged by the authors, is the persistence of spontaneous activity at these synapses, which could still impact the organization of these regions.

Comments on revisions:

The authors have addressed all of my previous comments.

# Referee report: Reviewer #2 (Public review)

**DOI:** 10.7554/eLife.107276.3.sa2

Summary:

This study examines whether the localization of endocytic proteins to presynaptic periactive zones depends on synaptic activity or active zone scaffolds. Using genetic and pharmacological perturbations in both *Drosophila* and mouse neurons, the authors show that key endocytic proteins remain localized to periactive zones even when evoked release or active zone architecture is disrupted. While the findings are largely negative, the study is methodologically solid and provides useful constraints for current models of synaptic vesicle recycling.

Strengths:

The experimental design is careful and systematic, spanning both fly and mammalian systems. The use of advanced genetic models, including Liprin-α quadruple knockout mice, is a notable strength. High-resolution imaging approaches (STED, Airyscan) are appropriately applied to assess nanoscale organization. The study clarifies that strict activity dependence of endocytic recruitment may not be a general principle.

Weaknesses (largely addressed in revision):

Several initial concerns have been satisfactorily addressed in the revised manuscript. In particular, the inclusion of EndoA/Dap160 experiments and the expanded discussion improve the work. Some limitations remain, including the reliance on Tetanus toxin at the *Drosophila* NMJ, which does not fully abolish presynaptic fusion, and the still limited insight into the mechanistic basis of periactive zone organization. The biological interpretation of small changes in protein levels upon silencing also remains somewhat unclear.

Comments on revisions:

I thank the authors for the careful revision of the manuscript. The additional experiments, in particular the inclusion of EndoA and Dap160 at the *Drosophila* NMJ, as well as the extended discussion of limitations, are appreciated and address important points raised in the first round.

While the principal conclusions of the study remain unchanged, and the manuscript is still largely based on negative results, I find that the authors now present these data in a more balanced and transparent manner. The discussion of activity-dependence is improved and more nuanced, especially with regard to possible contributions of spontaneous release and homeostatic effects.

In my opinion, despite the mostly negative nature of the findings, the work provides a valuable and relevant contribution, as it defines important constraints on current models of periactive zone organization. The study is technically strong, carefully executed, and systematically performed across different model systems.

Overall, the revised manuscript is clearly improved and represents a solid and well-executed piece of work that will be of interest to the field.

# Referee report: Reviewer #3 (Public review)

**DOI:** 10.7554/eLife.107276.3.sa3

Summary:

This study examines how synaptic endocytic zones are positioned using a combination of cultured neurons and the *Drosophila* neuromuscular junction. The authors test whether neuronal activity, active zone assembly, or liprin-α function is required to localize endocytic zone markers, including Dynamin, Amphiphysin, Nervous Wreck, PIPK1γ, and AP-180. None of the manipulations tested caused a coordinated disruption in the localization or abundance of these markers, leading to the conclusion that endocytic zones form independently of synaptic activity and active zone scaffolds.

Strengths:

The work is systematic and carefully executed, using multiple manipulations and two complementary model systems. The authors consistently examine multiple molecular markers, strengthening the interpretation that endocytic zone positioning is robust to changes in activity and structural assembly.

Weaknesses:

The main limitation is that the study does not test whether the methods used are sensitive enough to detect subtle functional disruption, and no condition tested produces clear disorganization of the endocytic zone. As a result, the conclusion that these zones assemble independently is supported by negative data, without a strong positive control for disassembly or mislocalization.

This paper addresses a longstanding question in synaptic biology and provides a well-supported boundary on the types of mechanisms that are likely to govern endocytic zone localization. The conclusions are well justified by the data, though additional evidence would be needed to define the assembly mechanism itself.

Comments on revisions:

The authors responded to the initial review with care. They both revised the manuscript and conducted new experiments to address each reviewer's concern. The responses to the review were effective, and I think that the revised manuscript provides significant new insights. In my view, it does not require additional revisions.

# Author comment: Author response

**DOI:** 10.7554/eLife.107276.3.sa4

**Authors:** Javier Emperador-Melero, Steven J Del Signore, Kevin M De León González, Pascal S Kaeser, Avital Adah Rodal

**Affiliations:** Harvard Medical School Boston United States; Brandeis University Waltham United States; Brandeis University Waltham United States; Harvard Medical School Boston United States; Brandeis University Waltham United States

The following is the authors’ response to the original reviews.

We thank the reviewers for their careful consideration of our work and constructive comments. We are glad that reviewers appreciated the rigor and value of our work. In response to the reviewer comments we have made the following changes:

(1) Addition of new experiments on EndoA localization at the *Drosophila* NMJ (Fig. 2).

(2) Addition of new experiments on Dap160 localization at the *Drosophila* NMJ (Fig. 2).

(3) Addition of new experiments to validate Dynamin, Dap160 and EndoA antibodies (Fig. 2 – figure supplement 1).

(4) Assessment of the activity-dependence of EndoA and Dap160 localization at the *Drosophila* NMJ (Fig. 3).

(5) Assessment of the liprin-dependence of EndoA and Dap160 localization at the *Drosophila* NMJ (Fig. 8).

(6) Addition of a limitations section to the discussion to directly address that spontaneous release was not fully ablated in our studies and might contribute to recruitment.

(7) Addition of an outlook to the same section on what experimental avenues could address the limitations in the future.

**Public Reviews:**

**Reviewer #1 (Public review):**

Summary:

In this manuscript, Emperador-Melero et al. seek to determine whether recruitment of endocytic machinery to the periactive zone is activity-dependent or tethered to delivery of active zone machinery. They use genetic knockouts and pharmacological block in two model synapses - cultured mouse hippocampal neurons and *Drosophila* neuromuscular junctions - to determine how well endocytic machinery localizes after chronic inhibition or acute depolarization by super-resolution imaging. They find that acute depolarization in both models has minimal to no effect on the localization of endocytic machinery at the periactive zone, suggesting that these proteins are constitutively maintained rather than upregulated in response to transient activity. Interestingly, chronic inhibition slightly increases endocytic machinery levels, implying a potential homeostatic upregulation in preparation for rebound depolarization. Using genetic knockouts, the authors show that localization of endocytic machinery to periactive zones occurs independently of proper active zone assembly, even in the absence of upstream organizers like Liprin-α. Overall, they propose that the constitutive deployment of endocytic machinery reflects its critical role in facilitating rapid and reliable membrane internalization during synaptic functions beyond classical endocytosis, such as regulation of the exocytic fusion pore and dense-core vesicle fusion. Although many experiments reveal limited changes in the localization or abundance of endocytic machinery, the findings are thorough, and data substantially support a model in which endocytic components are organized through a pathway distinct from that of the active zone. This work advances our understanding of synaptic dynamics by supporting a model in which endocytic machinery is constitutively recruited and regulated by distinct upstream organizers compared to active zone proteins. It also highlights the utility of super-resolution imaging across diverse synapse types to uncover functionally conserved elements of synaptic biology.

We thank the reviewer for the positive assessment of our study.

Strengths:

The study's technical strengths, particularly the use of super-resolution microscopy and rigorous image analyses developed by the group, bolster their findings.

We thank the reviewer for highlighting the technical strength of our work.

Weaknesses:

One notable limitation, however, is the absence of interrogation of endocytic proteins previously suggested to be recruited in an activity-dependent manner, in particular, endophilin.

We thank the reviewer for the suggestion. We have added experiments to assess the localization of two more proteins at *Drosophila* NMJs. These proteins are EndoA and Dap160, both of which have been reported to traffic between the synaptic vesicle cloud and the plasma membrane in response to stimulation [1-3]. In line with these studies, we observed that EndoA and Dap160 partially co-localize with a synaptic vesicle marker and with a periactive zone marker, indicating localization to both compartments (Fig. 2). However, neither high frequency stimulation nor expression of TeNT changed the levels or the distribution of these two proteins at the periactive zone (Fig. 3). Similarly, the deployment of these proteins at the periactive zone at the *Drospophila* NMJ was not dependent on the active zone scaffold Liprin-α (Fig. 8). Our data indicate that deployment of EndoA and Dap160 to the periactive zone does not require evoked synaptic activity.

We believe that there are multiple plausible explanations for our findings compared to previous work on Endophilin, which we discuss on lines 407-410: “Increased synaptic enrichment was also observed for Endophilin at nematode NMJs in mutants with disrupted exocytosis (Bai et al., 2010). We do not see such large shifts in Endophilin following similar manipulations, which might reflect distinct synaptic architectures in the *C. elegans* dorsal cord versus *Drosophila* NMJ terminals.” Further, this study finds that a plasma membrane-tethered Endophilin strongly colocalizes with endocytic machinery and largely rescues function. This suggests that the plasma membrane is the primary functional compartment for Endophilin. Together with our work, we conclude that these data suggest that Endophilin constitutively, but not completely, localizes to the periactive zone.

**Reviewer #2 (Public review):**

Summary:

This study examines whether the localization of endocytic proteins to presynaptic periactive zones depends on synaptic activity or active zone scaffolds. Using a combination of genetic and pharmacological perturbations in *Drosophila* and mouse neurons, the authors show that proteins such as Dynamin, Amphiphysin, AP-180, and others are still recruited to periactive zones even when evoked release or active zone architecture is disrupted. While the results are mostly negative, the study is methodologically solid and contributes to a more nuanced understanding of synaptic vesicle recycling machinery.

We thank the reviewer for deeming our work solid and for highlighting its importance for the field.

Strengths:

(1) The experimental design is careful and systematic, covering both fly and mammalian systems.

(2) The use of advanced genetic models (e.g., Liprin-α quadruple knockout mice) is a notable strength.

(3) High-resolution imaging (STED, Airyscan) is well used to assess spatial localization.

(4) The findings clarify that certain core assumptions - such as strict activity dependence of endocytic recruitment - may not hold universally.

We thank the reviewer for pointing out these strengths.

Weaknesses:

(1) The study would benefit from a clearer positive control to demonstrate activity-dependent recruitment (e.g., Endophilin).

We have added experiments to measure the localization of Endophilin, a protein previously reported to localize to the synaptic vesicle cloud [1], in *Drosophila* NMJs (Figs. 2 and 3). We observed that EndoA localized both to the synaptic vesicle cloud and to the periactive zone area. While stimulation did not enhance levels in either compartment, this outcome is not inconsistent with shuttling of protein between compartments during activity. Nevertheless, our data support a model in which EndoA, like the other tested endocytic proteins, is present at the periactive zone at rest.

(2) The reliance on Tetanus toxin in the *Drosophila* NMJ experiments in my eyes is a limitation, as it does not block all presynaptic fusion events; this should be discussed more directly.

We agree with the point of the reviewer. To more directly discuss it, we have included a “Limitations and Outlook” section in the revised version. We state that “conclusions that can be drawn on the roles of spontaneous release in periactive zone assembly remain limited” (lines 514-515). We further state that, while the manipulations that we included result in decreased spontaneous release, “it is possible that the remaining spontaneous release supports periactive zone assembly” (518-519) and that “Future studies might test manipulations with strong effects on miniature release including those affecting SNARE proteins and their regulators, with the caveat that these manipulations might have effects on upstream trafficking and in some cases on cell survival (Kaeser and Regehr, 2014; Santos et al., 2017).” (519-523).

(3) The potential role of Dynamin in organizing other periactive zone proteins is not addressed and could be an important next step.

We agree with the reviewer that this is an interesting possibility. On lines 454-455, we make the broad point that “interactions between endocytic proteins may further contribute to the anchoring of this apparatus”, and on lines 459-460, we specifically suggest a role for Dynamin by stating that “perturbing interactions between Dynamin-1 and Endophilin-A1 increases the distance between these proteins (Imoto et al., 2024), suggesting their binding has a scaffolding function.”

(4) Some small changes in protein levels upon silencing are reported; their biological meaning (e.g., compensation vs. variability) is not fully clarified.

These changes might include homeostatic adaptations. In the revised version of the manuscript, this is addressed on lines 135-137 and 405-407. We think it is overall difficult to assign biological meaning to small-magnitude changes, and chose to highlight the main point that there are no large-magnitude changes.

(5) While alternative organizing mechanisms (actin, lipids, adhesion molecules) are mentioned, a more forward-looking discussion of how to test these models would be helpful.

Following the reviewer’s suggestion, we have added an outlook section to the discussion where we provide suggestions for future studies (lines 510-543).

(6) The authors should consider including, or at least discussing, a well-established activity-dependent endocytic protein (e.g., Endophilin) as a positive control to help contextualize the negative findings.

We have included new experiments on EndoA at the fly neuromuscular junction (Fig. 2, Fig. 3, Fig. 8, Fig. 3 – figure supplement 1) and have added appropriate discussion of these findings as outlined above.

**Reviewer #3 (Public review):**

Summary:

This study examines how synaptic endocytic zones are positioned using a combination of cultured neurons and the *Drosophila* neuromuscular junction. The authors test whether neuronal activity, active zone assembly, or liprin-α function is required to localize endocytic zone markers, including Dynamin, Amphiphysin, Nervous Wreck, PIPK1γ, and AP-180. None of the manipulations tested caused a coordinated disruption in the localization or abundance of these markers, leading to the conclusion that endocytic zones form independently of synaptic activity and active zone scaffolds.

We thank the reviewer for reviewing our work.

Strengths:

The work is systematic and carefully executed, using multiple manipulations and two complementary model systems. The authors consistently examine multiple molecular markers, strengthening the interpretation that endocytic zone positioning is robust to changes in activity and structural assembly.

We thank the reviewer for pointing out these strengths.

Weaknesses:

The main limitation is that the study does not test whether the methods used are sensitive enough to detect subtle functional disruption, and no condition tested produces clear disorganization of the endocytic zone. As a result, the conclusion that these zones assemble independently is supported by negative data, without a strong positive control for disassembly or mislocalization.

We are confident that our methods are sensitive enough to detect changes within synaptic compartments. First, for mouse neurons assessed with STED microscopy, we have demonstrated that we can distinguish between the N- and the C-termini of the presynaptic protein Bassoon, which are positioned only a few tens of nanometers apart [4]. We have subsequently been consistently able to resolve the localization of pre- and postsynaptic proteins that also localize a few tens of nanometers apart and have established that genetic manipulations of active zone proteins induce detectable disruptions as assessed by STED microscopy [4-12]. Given that the periactive zone is larger than the distances that we can resolve, we are confident that we can detect changes in this area with enough sensitivity. Second, for *Drosophila* NMJs, we use a carefully validated workflow that allows assessing the distribution of periactive zone proteins and can detect subtle changes [13]. Unfortunately, there are no known manipulations that lead to periactive zone disassembly that could serve as a positive control, which reflects the little knowledge available in this field. We acknowledge that there may be subtle changes in protein localization that escape the resolution of our microscopy methods or experimental design, but this would not undermine the conclusion that the periactive zone remains assembled across the manipulations that we have tested. Overall, none of the manipulations we test induces a detectable disruption of the periactive zone. Naturally, we cannot exclude milder effects and have added a limitations section to discuss this possibility and some of the subtle changes we observe.

This paper addresses a longstanding question in synaptic biology and provides a well-supported boundary on the types of mechanisms that are likely to govern endocytic zone localization. The conclusions are well justified by the data, though additional evidence would be needed to define the assembly mechanism itself.

We thank the reviewer for the support of the conclusion of our study.

**Recommendations for the authors:**

**Reviewing Editor Comments:**

This is a rigorous study that, while presenting largely negative data, delimitates the processes that control peri-active zone organization. In addition to the interpretive and technical comments below, we encourage the authors to consider extending this study in two areas. First, examining the activity-dependence of Endophilin, and perhaps other factors, being recruited to the PAZ, where previous research has indicated a positive role for activity. Second, further characterization of the role of miniature release events in potentially contributing to PAZ organization. Overall, this was a rigorous and well-executed study.

We thank the reviewing editor for this positive assessment of our work.

**Reviewer #1 (Recommendations for the authors):**

(1) The rationale for comparing chronic inhibition to acute depolarization could be more clearly articulated. While this approach may be grounded in prior studies, the physiological consequences of chronic silencing differ markedly from those of transient activity, and these distinctions should be more explicitly addressed in the interpretation of results. For example, might lower intensity, chronic stimulation be a better comparison? Since fixation takes place immediately after stimulation, the time window to capture changes in protein recruitment may be curtailed.

We thank the reviewer for this comment. The introduction of the manuscript now includes a rationale on lines 110-112. By inhibiting evoked synaptic vesicle fusion throughout the lifespan of neurons, we assessed whether this process is necessary for periactive zone assembly and concluded that it is not a requirement. By acutely depolarizing neurons with 50 mM KCl or with a 40 Hz train of action potentials, we were able to test whether synaptic vesicle fusion triggers the rapid recruitment of endocytic proteins to the periactive zone and concluded that this is not the case for most of the endocytic proteins that we studied. While these results indicate that a constitutive pathway must exist to assemble the periactive zone, we remain agnostic as to whether stimulation paradigms not tested in our study can enhance the deployment of endocytic proteins, especially over long periods of time. This may be the case for low, chronic stimulation, as suggested by the reviewer. We clarify these limitations on a “limitations and outlook” section of the discussion (lines 510-543).

(2) Amphiphysin stood out as the only protein showing a notable change in opposite directions under either active zone protein knockout/blockers and Liprin-α knockout. Given the predominance of negative results, it would be valuable to devote more discussion to why Amphiphysin behaves differently. What functional role might it play in this context that sets it apart from other endocytic components?

As suggested by the reviewer, we have extended the discussion on Amphiphysin. One possibility why Amphiphysin may respond differently to different genetic manipulations or changes in stimulation is that different endocytic proteins might belong to different endocytic submachineries. This is addressed on lines 421-424. On lines 444-449, we further discuss the subtle decrease in the levels of Amphiphysin and AP-180 in Liprin-α mutants. We suggest that the actin cytoskeleton may be the link between the active zone and the endocytic apparatus, and that this link may be partially disrupted in Liprin-α mutants. Overall, we note that Amphiphysin is still localized to the periactive zone at rest, and hence that it fits with the overall model of constitutive deployment that we propose.

(3) The claim of activity-independence may need to be nuanced. Although the data suggest no recruitment in response to acute stimulation, the subtle changes following chronic inhibition complicate this interpretation, especially when considering redundancy. If activity-dependence is considered bidirectional, these findings might reflect a more complex regulatory mechanism. The interpretation in lines 188-190 more accurately captures this complexity than earlier generalizations.

We agree with the reviewer that the dependence on activity should be discussed in a nuanced fashion. We have scrutinized the manuscript on this point and state throughout that recruitment is independent of evoked activity and not necessarily of any kind of activity. We believe that this interpretation is accurate because evoked release of neurotransmitter was ablated by the pharmacological and genetic manipulations that we used. Furthermore, we have included a “Limitations of the study” section in the discussion where we openly address that spontaneous fusion of synaptic vesicles cannot be ruled out as a potential mechanism to sustain periactive zone assembly (lines 514-523). Finally, we have expanded on the complexity of periactive zone assembly relative to activity. In particular, homeostasis may contribute to increased levels of endocytic proteins upon chronic blockade of evoked transmission (lines 404-406).

(4) Given published work on endophilin's role in activity-dependent endocytic recruitment, adding endophilin (at least in the *Drosophila* NMJ experiments) would be highly informative.

We thank the reviewer for the suggestion. We have added experiments to assess the localization of two more proteins at *Drosophila* NMJs. These proteins are EndoA and Dap160, both of which have been reported to traffic between the synaptic vesicle cloud and the plasma membrane in response to stimulation [1-3]. In line with these studies, we observed that EndoA and Dap160 partially co-localize with a synaptic vesicle marker and with a periactive zone marker, indicating localization to both compartments (Fig. 2). However, neither high frequency stimulation nor expression of TeNT changed the levels or the distribution of these two proteins at the periactive zone (Fig. 3). Similarly, the deployment of these proteins at the periactive zone at the *Drosophila* NMJ was not dependent on the active zone scaffold Liprin-α (Fig. 8). Our data indicate that deployment of EndoA and Dap160 to the periactive zone does not require evoked synaptic activity.

We believe that there are multiple plausible explanations for these findings compared to previous work on Endophilin [3], which we discuss on lines 407-410:

“Increased synaptic enrichment was also observed for Endophilin at nematode NMJs in mutants with disrupted exocytosis (Bai et al.,2010). We do not see such large shifts in Endophilin following similar manipulations, which might reflect distinct synaptic architectures in the *C. elegans* dorsal cord vs *Drosophila* NMJ terminals.” Further, this study finds that a plasma membrane-tethered Endophilin strongly colocalizes with endocytic machinery and largely rescues function. This suggests that the plasma membrane is the primary functional compartment for Endophilin. Together, all data are compatible with a model in which Endophilin constitutively, but not completely, localizes to the periactive zone.

(5) Line 57 might have a typo in the citation.

We thank the reviewer for pointing this out. The citations now include: Bai et al., 2010; Jiang et al., 2024; Koh et al., 2007; Winther et al., 2013 and Winther et al. 2015. Please note that these two last citations are grouped as Winther et al. 2013, 2015 following our formatting style.

(6) Line 208 might be missing a citation that justifies parameters.

In the revision, this information is discussed on lines 222-224, where we cite our prior work describing these data: “Each unit is divided into ‘mesh’ and ‘core’ regions, where the periactive zone mesh is a ~175 nm wide area localized at ~330 nm from the center, and the ‘core’ region is the interior to this mesh (Del Signore et al., 2023)”.

**Reviewer #2 (Recommendations for the authors):**

(1) Please consider including, or at least discussing, a well-established activity-dependent endocytic protein (e.g., Endophilin) as a positive control to help contextualize the negative findings.

We thank the reviewer for the suggestion. We have added experiments to assess the localization of two more proteins at *Drosophila* NMJs. These proteins are EndoA and Dap160, both of which have been reported to traffic between the synaptic vesicle cloud and the plasma membrane in response to stimulation [1-3]. In line with these studies, we observed that EndoA and Dap160 partially co-localize with a synaptic vesicle marker and with a periactive zone marker, indicating localization to both compartments (Fig. 2). However, neither high frequency stimulation nor expression of TeNT changed the levels or the distribution of these two proteins at the periactive zone (Fig. 3). Similarly, the deployment of these proteins at the periactive zone at the *Drosophila* NMJ was not dependent on the active zone scaffold Liprin-α (Fig. 8). Our data indicate that deployment of EndoA and Dap160 to the periactive zone does not require evoked synaptic activity.

We believe that there are multiple plausible explanations for our findings compared to previous work on Endophilin [3], which we discuss on lines 407-410: “Increased synaptic enrichment was also observed for Endophilin at nematode NMJs in mutants with disrupted exocytosis (Bai et al.,2010). We do not see such large shifts in Endophilin following similar manipulations, which might reflect distinct synaptic architectures in the *C. elegans* dorsal cord vs *Drosophila* NMJ terminals.” Further, this study finds that a plasma membrane-tethered Endophilin strongly colocalizes with endocytic machinery and largely rescues function. This suggests that the plasma membrane is the primary functional compartment for Endophilin. Together, all data are consistent with a model in which Endophilin constitutively, but not completely, localizes to the periactive zone.

(2) Expand the discussion of TeNT's limitations-specifically that it does not block spontaneous fusion or alternative fusion pathways-and consider referencing more stringent tools (e.g., Botulinum toxins or SNARE mutants), even if they weren't used here.

Following the reviewer’s suggestion, we have included a “Limitations and Outlook” section in the revised version. We state that “conclusions that can be drawn on the roles of spontaneous release in periactive zone assembly remain limited” (lines 514-515). We further state that, while the manipulations that we included result in decreased spontaneous release, “it is possible that the remaining spontaneous release supports periactive zone assembly” (518-519) and that “Future studies might test manipulations with strong effects on miniature release including those affecting SNARE proteins and their regulators, with the caveat that these manipulations might have effects on upstream trafficking and in some cases on cell survival (Kaeser and Regehr, 2014; Santos et al., 2017)” (520-523).

(3) We encourage the authors to briefly discuss whether Dynamin might contribute to periactive zone structure beyond its role in membrane fission. Loss-of-function data could be particularly informative in future work.

We agree with the reviewer that this is an interesting possibility. On lines 454-455, we make the broad point that “interactions between endocytic proteins may further contribute to the anchoring of this apparatus”, and on lines 459-460, we specifically suggest a role for Dynamin by stating that “perturbing interactions between Dynamin-1 and Endophilin-A1 increases the distance between these proteins (Imoto et al., 2024), suggesting their binding has a scaffolding function.”

(4) Clarify the interpretation of increased endocytic protein levels upon chronic silencing - are these interpreted as homeostatic responses or experimental variability?

We suggest that these changes might include homeostatic adaptations. We note that this increase is of the same magnitude as the increase in active zone proteins following a similar pharmacological manipulation on lines 405-406, where we state that “a mechanism for this effect might be a homeostatic response (Wen and Turrigiano, 2024) similar in magnitude to the increase in active zone protein levels following activity blockade (Held et al., 2020).”

(5) The Discussion could be strengthened by sketching out more concrete experimental approaches to test candidate mechanisms (e.g., roles for actin, lipids, adhesion molecules) in organizing periactive zones.

The potential roles of the cell adhesion molecules (lines 430-440), cytoskeleton and lipids (442-452) are addressed in the discussion. Furthermore, following the reviewer’s suggestion, we have added the following statement (lines 541-543): “This work builds a foundation to assess alternative mechanisms and models of periactive zone assembly, including roles of the cytoskeleton, lipids, adhesion molecules, and intrinsic endocytic protein interactions”. We hope that the reviewer agrees that the discussion of our paper is not the right format to provide a concrete experimental plan for future work. In our view, the discussion should put the findings of our experiments in the context of the field.

**Reviewer #3 (Recommendations for the authors):**

(1) At a spine synapse, the endocytic zone is estimated to be between 100-200nm from the active zone. The focus of the author's analysis is largely outside of this region (0-150nm), raising the question of whether the area studied may be outside of the area affected by the manipulations made. While STED systems claim ~80 nm resolution, this is rarely achieved in practice, and the authors do not report the effective resolution of their system. Reporting the resolution achieved would address this issue. In addition, super-resolution imaging does not appear to have been used at the *Drosophila* NMJ. The authors should clarify whether resolution limitations influenced the choice of analysis region and whether their imaging approach is sufficient to detect changes in the endocytic zone.

We believe that it is unlikely that the relevant signals were missed. First, in mouse synapses, most signal corresponding to endocytic proteins was detected inside the selected region of interest. Our rationale to select the area was based on the fact that expanding the region analyzed would have reduced the sensitivity of our approach, as averaging over a larger area would dilute the signal. The resolution of our microscopy should not be a limitation either. In our previous work, we demonstrated that STED microscopy allows discriminating between the N- and the C-terminal termini of the presynaptic scaffold Bassoon, which are positioned only a few tens of nanometers apart [4]. This establishes that we can resolve differences at tens of nanometers in biological context, which is more relevant than the resolution measured with fluorescent beads (which we have repeatedly assessed to be ~80 nm laterally). Subsequently, we have also been consistently able to resolve the localization of pre- and postsynaptic proteins that also localize a few tens of nanometers apart [4-12]. Given that the periactive zone spans over a larger area than the distances that we can resolve experimentally in the examples above, we are confident that our measurements are sensitive enough to detect changes in this area.

Second, for *Drosophila* NMJs, the choice for the region of interest and the overall analysis was done following a workflow validated in our previous work [13]. This method analyzes both immediately adjacent and more distant regions from the active zone, and does not exclude any region based on distance from the active zone as described on lines 222-224: “Each unit is divided into ‘mesh’ and ‘core’ regions, where the periactive zone mesh is a ~175 nm wide area localized at ~330 nm from the center, and the ‘core’ region is the interior to this mesh (Del Signore et al., 2023).” In our previous study, we analyzed the distribution of periactive zone proteins at rest with STED microscopy and with Airyscan confocal microscopy. The resolution provided by Airyscan is reported to be ~175 nm in XY and ~400 nm in Z, which is sufficient to assess localization to the periactive zone compartment imaging methods and is not inferior to imaging methods previously used to report changes in the distribution of endocytic proteins; for examples, see [1,2]. In the revised manuscript, we have added new data measuring the levels and distribution of EndoA and Dap160 using STED microscopy (Figure 3 – figure supplement 1). The results acquired with STED microscopy and with Airyscan confocal microscopy are consistent with one another.

Overall, the accuracy of the imaging methods and analyses used in this study are sufficient to assess periactive zone structure given its size and organization.

(2) Interestingly, in a number of cases, the authors observe significant differences in endocytic markers (Figure 1q, 4k, 6k, 6r). However, little is made of these differences. The authors should provide more discussion of these changes and how they make sense of them alongside their claims of a lack of effect from their manipulations.

The reviewer raises a good point. We interpret these changes in two different ways. First, we suggest that changes observed in response to block of action potentials or disassembly of the active zone might be homeostatic. This is addressed on lines 135-137. Second, we discuss that the actin cytoskeleton may be the link between the active zone and the endocytic apparatus. Several active zone proteins interact with the actin cytoskeleton. One of them is Liprin-α. This interaction may explain the decrease in the level of Amphiphysin and AP-180 at the periactive zone in Liprin-α null neurons. This is addressed on lines 444-449. We hope that the reviewer agrees that overall, we should focus on the main conclusion that deployment of endocytic proteins persists over a number of manipulations and synapse types.

(3) The graphs in Figure 1c and 1g, 3g, 4c, 4e, 6c, and 6g do not appear to be identical. If the solid line represents the mean and the lighter color represents the distribution of these data, these data appear to be different from one another. It is surprising that these differences are not significant. What statistical tests were used to determine whether the differences in these graphs are not significant? Is the issue that a relatively now number of synapses were examined (30-60)? Did the authors conduct a power analysis?

We apologize if the display of our data and analyses was not clear. We do not perform statistical analyses on the line profiles. Instead, we perform it on two values that are extracted from line profiles. These values are (1) the distance between the peak intensity values of the protein of interest and the marker and (2) the peak intensity values. For example, in Figure 1, distances are quantified and statistically analyzed in panel j, and the peak levels are quantified and statistically analyzed in panel k. We have clarified this in the legend of current Figures 1, 4, 5, and 7.

(4) The authors clearly state that their experiments address the role of evoked activity in endocytic zone positioning, but they do not examine whether spontaneous vesicle fusion might play a role. Given the availability of *Drosophila* mutants that decrease (Doc2, Dunc-13) or increase (syt1) spontaneous release, this is a notable omission. Ideally, these mutants should be examined. And at a minimum, the authors should discuss whether spontaneous release could contribute to endocytic zone organization.

We agree with the reviewer that spontaneous fusion of synaptic vesicles may contribute to periactive zone organization. Many of the genetic manipulations that we used in mouse neurons result in a significant decrease in spontaneous release. This includes Ca_V_2 triple knockouts with a ~60% decrease in spontaneous fusion [10], RIM+ELKS quadruple knockouts with a ~70% decrease in spontaneous fusion [9] and Liprin-α quadruple knockouts with a ~50% decrease in spontaneous fusion [7]. We cannot rule out that the spontaneous release that is left is sufficient to mediate assembly functions. The conclusive way to address this possibility is using a manipulation that ablates spontaneous release without altering other pathways. However, to our knowledge, this is not available. The manipulations suggested by the reviewer might suffer from similar limitations, as they would change the frequency of spontaneous release without fully ablating it, and they would also affect evoked release. We have included a limitations section in the discussion where we address this (lines 514-523), specifically stating “conclusions that can be drawn on the roles of spontaneous release in periactive zone assembly remain limited. While many of the manipulations used here, including Ca_V_2 knockout (Held et al., 2020), RIM+ELKS knockout (Tan et al., 2022; Wang et al., 2016) and Liprin-α knockout (Emperador-Melero et al., 2024) in hippocampal neurons, and TeNT expression in fly NMJs (Sweeney et al.,1995) , result in 50% to 70% decreased spontaneous release rates, it is possible that the remaining spontaneous release supports periactive zone assembly. Future studies might test manipulations with strong effects on miniature release including those affecting SNARE proteins and their regulators, with the caveat that these manipulations might have effects on upstream trafficking and in some cases on cell survival (Kaeser and Regehr, 2014; Santos et al., 2017).” We hope that the reviewer agrees that assessing these mutants should be a topic of future studies, given that we already test many mutants in the paper.

(5) In Figures 1 and 6, the authors assess presynaptic protein localization in cultured neurons, but it is unclear whether these are synaptic sites. Many presynaptic proteins traffic together and can accumulate at sites lacking postsynaptic specializations. The authors should validate that the observed spatial organization occurs at bona fide synapses, ideally by co-labeling with postsynaptic markers as done in Figure 4. If methods like these were used, providing more details on how synapses were identified and selected would be useful to the reader.

While we understand the reviewer’s point, we are confident that the structures analyzed are bona fide synapses for three reasons, as we have established before across many papers [4-8,10-12,17].

The diameter of the structures detected using the synaptic vesicle marker Synaptophysin aligns much more closely with the size of the large vesicle clusters found at presynaptic terminals than with that of a few transport vesicles.

In side-view synapses, the bar-like distribution of the active zone marker (Bassoon or Munc13-1) at one edge of the vesicle cloud indicates that active zone proteins are organized at one edge of the vesicle cluster—consistent with the architecture of synapses.

Synaptophysin is one of our key markers for detecting synapses. In our cultures, most of the Synaptophysin signal colocalizes with postsynaptic markers (either PSD-95 or Gephyrin), as we have established across many studies [4,7-12]. This indicates that the markers used here are sufficient to select synapses. Furthermore, the frequency at which synapses were identified using an active zone marker as the second marker was similar to that observed when using a postsynaptic marker, suggesting that we were not randomly including unrelated structures.

(6) Many of the images, particularly of the *Drosophila* NMJ, are of low quality and are shown in very small images. In addition, the quality of the images throughout the paper makes it difficult to assess the author's analysis and results. The authors should provide larger, higher-quality images that show examples of the means for each of the examples shown. This is an issue for most of the figures, but is particularly prominent in the dNMJ. A minor additional point is that the authors should be clear whether the dNMJ images are collected at super-resolution or using a conventional microscope.

We believe that the quality of our images is sufficient for the assessments made for the following reasons:

These images were acquired with enough spatial resolution to assess levels at the PAZ as discussed in response to this reviewer’s first comment. In our previous work, we used images acquired at the same resolution and presented in the same manner for both mouse hippocampal synapses [6,7] and *Drosophila* NMJs [13,18]. In those previous studies, we drew conclusions at a similar level of detail as in the current study.

In our view, our representative images are not inferior in quality to other papers in the field addressing similar questions [1,2,19,20].

We have selected sample images based on the quantified mean values per condition. Hence, we strived to select panels that are objectively representative regarding the quantified parameters.

We have specified microscopy methods in the figure legends. Specifically, for *Drosophila* NMJs, we used Airyscan confocal microscopy and STED microscopy. For each experiment, it is now stated which microscopy method was used in the corresponding legend.

References:

(1) Winther, Å. M. E. et al. An Endocytic Scaffolding Protein together with Synapsin Regulates Synaptic Vesicle Clustering in the *Drosophila* Neuromuscular Junction. J Neurosci 35, 14756–14770 (2015).

(2) Winther, Å. M. E. et al. The dynamin-binding domains of Dap160/intersectin affect bulk membrane retrieval in synapses. J Cell Sci 126, 1021–1031 (2013).

(3) Bai, J., Hu, Z., Dittman, J. S., Pym, E. C. G. & Kaplan, J. M. Endophilin functions as a membrane-bending molecule and is delivered to endocytic zones by exocytosis. Cell 143, 430–441 (2010).

(4) Wong, M. Y. et al. Liprin-alpha3 controls vesicle docking and exocytosis at the active zone of hippocampal synapses. Proc Natl Acad Sci U S A 115, 2234–2239 (2018).

(5) Emperador-Melero, J., de Nola, G. & Kaeser, P. S. Intact synapse structure and function after combined knockout of PTPδ, PTPσ, and LAR. Elife 10, (2021).

(6) Emperador-Melero, J. et al. PKC-phosphorylation of Liprin-α3 triggers phase separation and controls presynaptic active zone structure. Nat Commun 12, 3057 (2021).

(7) Emperador-Melero, J. et al. Distinct active zone protein machineries mediate Ca2+ channel clustering and vesicle priming at hippocampal synapses. Nature Neuroscience 2024 1–15 (2024) doi:10.1038/s41593-024-01720-5.

(8) Tan, C., Wang, S. S. H., de Nola, G. & Kaeser, P. S. Rebuilding essential active zone functions within a synapse. Neuron 110, 1498-1515.e8 (2022).

(9) Wang, S. S. H. et al. Fusion Competent Synaptic Vesicles Persist upon Active Zone Disruption and Loss of Vesicle Docking. Neuron 91, 777–791 (2016).

(10) Held, R. G. et al. Synapse and Active Zone Assembly in the Absence of Presynaptic Ca(2+) Channels and Ca(2+) Entry. Neuron 107, 667-683.e9 (2020).

(11) Chin, M. & Kaeser, P. S. The intracellular C-terminus confers compartment-specific targeting of voltage-gated calcium channels. Cell Rep 43, 114428 (2024).

(12) Nyitrai, H., Wang, S. S. H. & Kaeser, P. S. ELKS1 Captures Rab6-Marked Vesicular Cargo in Presynaptic Nerve Terminals. Cell Rep 31, 107712 (2020).

(13) Del Signore, S. J., Mitzner, M. G., Silveira, A. M., Fai, T. G. & Rodal, A. A. An approach for quantitative mapping of synaptic periactive zone architecture and organization. Mol Biol Cell 34, (2023).

(14) Sweeney, S. T., Broadie, K., Keane, J., Niemann, H. & O’Kane, C. J. Targeted expression of tetanus toxin light chain in *Drosophila* specifically eliminates synaptic transmission and causes behavioral defects. Neuron 14, 341–351 (1995).

(15) Kaeser, P. S. & Regehr, W. G. Molecular mechanisms for synchronous, asynchronous, and spontaneous neurotransmitter release. Annu Rev Physiol 76, 333–363 (2014).

(16) Santos, T. C., Wierda, K., Broeke, J. H., Toonen, R. F. & Verhage, M. Early Golgi Abnormalities and Neurodegeneration upon Loss of Presynaptic Proteins Munc18-1, Syntaxin-1, or SNAP-25. Journal of Neuroscience 37, 4525–4539 (2017).

(17) de Jong, A. P. H. et al. RIM C2B Domains Target Presynaptic Active Zone Functions to PIP2-Containing Membranes. Neuron 98, 335-349.e7 (2018).

(18) Del Signore, S. J. et al. An autoinhibitory clamp of actin assembly constrains and directs synaptic endocytosis. Elife 10, (2021).

(19) Imoto, Y. et al. Dynamin 1xA interacts with Endophilin A1 via its spliced long C-terminus for ultrafast endocytosis. EMBO Journal https://doi.org/10.1038/S44318-024-00145-X

(20) Imoto, Y. et al. Dynamin is primed at endocytic sites for ultrafast endocytosis. Neuron 110, 2815-2835.e13 (2022).
